# The orphan nuclear receptor TLX: an emerging master regulator of cross-talk between microglia and neural precursor cells

**DOI:** 10.1042/NS20180208

**Published:** 2019-06-06

**Authors:** Paul J. Lucassen, Anne-Marie van Dam, Prasanna Kandel, Pascal Bielefeld, Aniko Korosi, Carlos P. Fitzsimons, Mirjana Maletic-Savatic

**Affiliations:** 1 Swammerdam Institute for Life Science, Center for Neuroscience, University of Amsterdam, Science Park 904, Amsterdam 1098 XH, The Netherlands; 2 Department of Anatomy and Neurosciences, Amsterdam University Medical Center, Vrije Universiteit Amsterdam, Amsterdam, The Netherlands; 3 Baylor College of Medicine and Jan and Dan Duncan Neurological Research Institute at Texas Children’s Hospital, Houston, TX, U.S.A.

**Keywords:** hippocampus, microglia, neurogenesis, TLX

## Abstract

Neuroinflammation and neurogenesis have both been the subject of intensive investigation over the past 20 years. The sheer complexity of their regulation and their ubiquity in various states of health and disease have sometimes obscured the progress that has been made in unraveling their mechanisms and regulation.

A recent study by Kozareva et al. (*Neuronal Signaling* (2019) **3**), provides evidence that the orphan nuclear receptor TLX is central to communication between microglia and neural precursor cells and could help us understand how inflammation, mediated by microglia, influences the development of new neurons in the adult hippocampus.

Here, we put recent studies on TLX into the context of what is known about adult neurogenesis and microglial activation in the brain, along with the many hints that these processes must be inter-related.

## Commentary

The orphan nuclear receptor TLX, first identified in retinal photoreceptors 20 years ago [1,2], has been studied mostly for its role in regulating the ongoing creation of new neurons in the adult hippocampus [3–5]. This adult neurogenesis occurs in at least several mammalian species, including humans [6–11] and has been implicated in learning, memory, stress regulation and maintaining emotional resilience [6,7,12–14]. There has been considerable interest in harnessing neurogenesis to treat disorders ranging from depression to Alzheimer’s [6,8,10,12,15] but much remains to be learned about how the process is regulated and why it sometimes fails. In a recent paper in *Neuronal Signaling*, Kozareva et al. [16] made a significant contribution to our understanding of neurogenesis by demonstrating a new role for TLX in mediating communication between the neural stem cells and neighboring microglia.

The birth, migration, differentiation and functional integration of the newborn neurons into the adult hippocampal circuitry is influenced by various factors: exercise and intellectual/social stimulation promote neurogenesis, whereas stress, loneliness, and inflammation suppress it [6,7,13,17–19]. Also diet, specific nutrients and sleep (deprivation) modify neurogenesis, often in an age-specific manner [20–24]. Certain microRNAs and transcription factors can influence specific stages of the neurogenic process and modulate the proliferation of neural stem cells or their subsequent survival [25–30]. While one microRNA can regulate many different target genes, each microRNA can in turn be regulated by various other genes or regulatory factors.

When regulation is this complex and interdependent, there are often ‘master’ or ‘upstream’ factors that exert a higher level of control. This appears to be the function of the orphan nuclear receptor TLX (Nr2e1), whose role in hippocampal neurogenesis has been increasingly appreciated over the past decade [3–5,31,32]. TLX is expressed in both neurogenic niches (the subventricular zone of the ventricular walls and the subgranular zone of the dentate gyrus), specifically in the neuronal precursor cells (NPCs), which it maintains in a proliferative state by preventing them from undergoing ectopic differentiation. Consistent with this functional role, TLX knockout mice display impaired neurogenic responses, diminished hippocampal volume, impaired long-term potentiation in the dentate gyrus and deficits in hippocampal memory and related behaviors [3–5,32,35].

Interestingly, TLX has also been linked to neuro-inflammatory changes and to microglia, the brain’s immune cells [36–39]. Microglia contribute to synaptic pruning and phagocytic clearance of subsets of the adult-born cells [40,41]. They show specific morphological changes with age [42–44] and the neuro-inflammatory mediators they produce during this process and in related diseases impair hippocampal function and suppress neurogenesis [18,19,41,45,46]. Specifically, the pro–inflammatory cytokine interleukin–1β (IL–1β), which is induced in microglia after peripheral inflammation [47,48], suppresses both hippocampal NPC proliferation and TLX expression [49–53]. IL–1β also induces a range of transcriptional changes that are regulated by TLX [51], but in the absence of TLX, microglia become activated and induce hippocampal inflammation [50,53,54].

To delineate pathways of communication between TLX and microglia, Kozareva et al. [16] assessed microRNA expression patterns of known up– or downstream signaling molecules of TLX in the hippocampus of mice lacking CX3CR1, a microglially expressed receptor for the chemokine CX3CL1 (fractalkine) that is released by adult neurons [55,56]. The authors show that in these knockout mice, expression of TLX and its downstream, but not upstream, targets, were selectively reduced. Instead, there is an up–regulation of the TLX repressor MIR–378 and increases in levels of the target genes bone morphogenic protein 4 (BMP4) and PTEN. However, no change in other miRNAs, such as miR–9, miR–137, miR–let7d or miR–let7b, which suppress TLX both *in vitro* and *in vivo*, were observed. Therefore, the reduction in TLX in CX3CR1–KO mice must be independent of both the TLX–miR–let7b regulatory loop and the TLX–miR–9 feedback pathway. This is consistent with the concept that an absence of CX3CR1 could down–regulate TLX via a self–repression mechanism [35,57–60] and positions TLX as a potential target or co–regulator of the CX3CR1/CX3CL1 pathway. The concomitant reduction in hippocampal neurogenesis observed in CX3CR1 knockout mice may thus result from activation of TLX–suppressing signaling pathways that inhibit activation of quiescent NSCs and maintain them in a non–proliferative state through PTEN signaling. By identifying these factors, Kozareva et al. [16] extend the work of Ó’Léime et al. [50] to provide the pathways linking microglia activation and IL–1β increases to reductions in TLX and neurogenesis [42,51,52,60].

TLX overexpression increases neuronal precursor cell proliferation, hippocampal neurogenesis and enhances learning and memory [61]. Similar gain of function approaches could shed light on gliogenesis, and whether this also involves CX3CL1/CX3CR1–dependent mechanisms. Whereas Kozareva et al. [16] has studied whole hippocampal homogenates, a more specific analysis of the NSCs isolated from the dentate gyrus would be very informative. Similarly, future studies of the other neurogenic regions of the brain, such as the subventricular zone, will determine whether the microglia behave differently in this region. Given that TLX is involved in the transcriptional repression of BMP4, which is involved in astrogenesis, it would be of considerable interest to examine whether the reduction in hippocampal neurogenesis in CX3CR1 knockout mice is coupled to changes in hippocampal astrogenesis. Astrocytes can produce IL–1β in the central nervous system and thus may act as the ‘middle man’ in the cascade that suppresses neurogenesis as a result of CX3CR1 and/or TLX deficiency.

It will also be interesting to characterize the role of TLX in neurogenesis–microglia communication under conditions that are known to modulate neurogenesis and/or inflammation, such as chronic stress, aging, inflammation or disease [6,7,10,11,18,19,40]. The same applies for a possible role for TLX in the rapid clearance of apoptotic newborn cells, which is mediated by microglia [41], and it will be interesting to assess this under conditions of immune suppression, TLX targeting or knockdown, blocked or depleted microglia activity, or other environmental or pharmacological manipulations [17,44,62,63].

Studies further investigating the relationship between TLX and CX3CL1/CX3CR1 signaling will provide valuable information to understand the implications and functional roles of this master regulator. Mediators of neuro-inflammation are involved in brain diseases ranging from depression to Alzheimer’s, and a better understanding of the roles of TLX will provide important insights into the basic mechanisms of hippocampal plasticity and neurogenic–microglial cross-talk. Much remains to be learned in order to develop preventive or anti-inflammatory therapies for the hippocampal changes in these disorders, but studies like this are making clear progress toward this goal.

## Abbreviations

BMP4: bone morphogenic protein 4
IL-1β: interleukin 1β
NPC: neuronal precursor cell
